# A hybrid neural network model for predicting kidney disease in hypertension patients based on electronic health records

**DOI:** 10.1186/s12911-019-0765-4

**Published:** 2019-04-04

**Authors:** Yafeng Ren, Hao Fei, Xiaohui Liang, Donghong Ji, Ming Cheng

**Affiliations:** 1 Guangdong Collaborative Innovation Center for Language Research and Services, Guangdong University of Foreign Studies, Guangzhou, China; 2 School of Cyber Science and Engineering, Wuhan University, Wuhan, China; 3 School of Health Sciences, Wuhan University, Wuhan, China; 4 The First Affiliated Hospital, Zhengzhou University, Zhengzhou, China

**Keywords:** Disease prediction, Neural network, Electronic health records, Long short-term memory, Kidney disease

## Abstract

**Background:**

Disease prediction based on Electronic Health Records (EHR) has become one hot research topic in biomedical community. Existing work mainly focuses on the prediction of one target disease, and little work is proposed for multiple associated diseases prediction. Meanwhile, a piece of EHR usually contains two main information: the textual description and physical indicators. However, existing work largely adopts statistical models with discrete features from numerical physical indicators in EHR, and fails to make full use of textual description information.

**Methods:**

In this paper, we study the problem of kidney disease prediction in hypertension patients by using neural network model. Specifically, we first model the prediction problem as a binary classification task. Then we propose a hybrid neural network which incorporates Bidirectional Long Short-Term Memory (BiLSTM) and Autoencoder networks to fully capture the information in EHR.

**Results:**

We construct a dataset based on a large number of raw EHR data. The dataset consists of totally 35,332 records from hypertension patients. Experimental results show that the proposed neural model achieves 89.7% accuracy for the task.

**Conclusions:**

A hybrid neural network model was presented. Based on the constructed dataset, the comparison results of different models demonstrated the effectiveness of the proposed neural model. The proposed model outperformed traditional statistical models with discrete features and neural baseline systems.

## Background

In the modern society, people may suffer from all kinds of diseases, e.g., coronary heart disease, diabetes, hypertension, kidney disease, etc. More seriously, some people may be attacked by multiple diseases simultaneously. These diseases are often related to each other. Multiple associated diseases prediction is an important research topic in biomedical field, which aims to predict the prevalence of a target disease in the condition of the other certain disease that is already diagnosed.

Among these diseases, kidney disease is a worldwide public health issue. Many studies have been conducted for kidney disease risk analysis, and hypertension is commonly considered to be closely related for the development of kidney disease [1–7]. However, the risk factors that cause hypertension patients develop into kidney disease remain unclear.

**Fig. 1:**
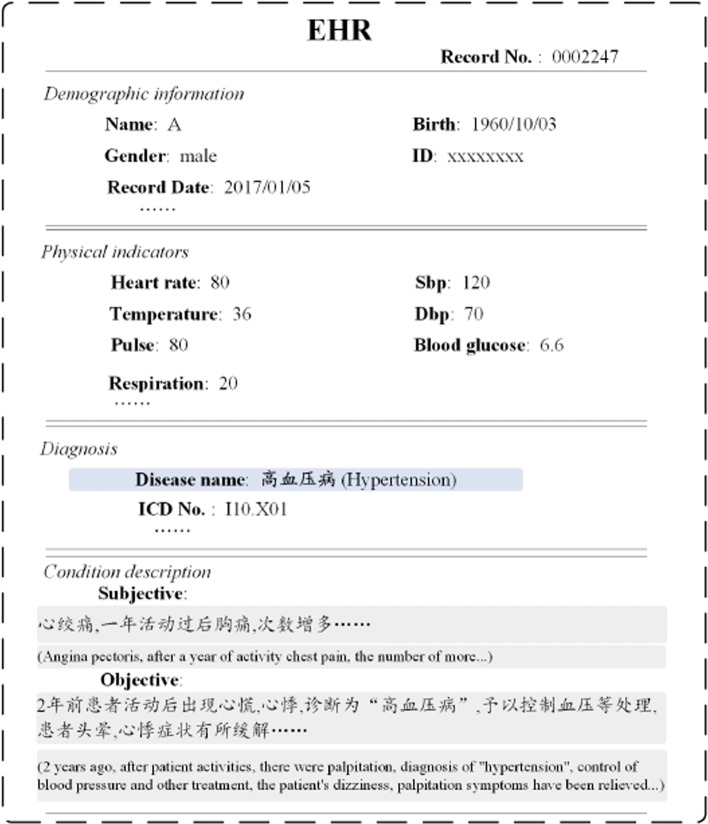
A piece of EHR from one hypertension patient

Electronic Health Record (EHR) usually contains two main information: textual description and discrete physical indicators. A piece of EHR are shown in Fig. 1. We can see that a patient is diagnosed with hypertension on January 5, 2017. Three months later, he is diagnosed with kidney disease. Given a patient who has been diagnosed with hypertension, this paper aims to predict the probability of the person to suffer from kidney disease.

In recent years, researchers begin to explore the task of the disease prediction by using machine learning techniques. Existing work mainly focuses on the prediction of one target disease [8–11]. For example, Jabbar et al., (2016) use random forest and chi square to predict heart disease [11]. Meanwhile, existing work mostly explores underlying molecular mechanisms of diseases [12–14]. Typically, Le and Dang (2016) propose a ontology-based disease similarity network for disease gene prediction [12]. However, little work is proposed for multiple associated diseases prediction. More recently, Chen et al., (2017) evaluate the risk factors that cause hypertension patients develop into coronary heart disease by using Logistic Regression (LR) model [15]. However, this model only uses the numerical physical indicators in EHR, which limits the performance of the task.

Recently, neural network models have been extensively used for text analysis tasks [16–18], achieving competitive results. Potential advantage of using neural networks for the disease prediction is that neural models use hidden layer for automatic feature combinations, which can capture complex semantic information that is difficult to express using traditional discrete manual features. This motivates a neural network model, which integrates the textual description information and physical indicators in EHR, for predicting kidney disease in hypertension patients.

In this paper, we first model the prediction problem as a binary classification task. Then, we construct a dataset based on a large amount of raw EHR data. Third, we build a hybrid neural network which incorporates Bi-directional Long Short Term Memory (BiLSTM) and Autoencoder network for the task. Here, BiLSTM is used for learning the textual features from textual description information. The Autoencoder network takes the numerical indicators as input for capturing important numerical cues. Experimental results show that the proposed neural model achieves the current best performance, significantly outperforming traditional discrete models and neural baseline systems. To our knowledge, our study is the first one for multiple associated diseases prediction task by using neural network.

## Related work

Disease prediction, especially the chronic diseases, has received more and more attention from researchers in the biomedical field [19–22]. Early researches mainly focus on the numerical factors including physical examination factors, laboratory test features, and demographic information. For example, Wilson et al., (1998) predicted the risk of coronary heart disease by using Logistic Regression model with an array of discrete factors [8]. The follow-up studies tried to estimate coronary heart disease by considering more non-traditional risk factors, in order to yield better performance [19, 23]. However, these work focuses on the prediction of single target disease. Meanwhile, these methods mainly use discrete models with hand-crafted features.

About ten years ago, researchers began to predict the disease risks from the genetic study and tried to find underlying molecular mechanisms of diseases [24–26]. For example, Wray et al., (2007) proposed to assess the genetic risk of a disease in healthy individuals based on dense genome-wide Single-Nucleotide Polymorphism (SNP) panels [26]. More recently, some researches explored the genes associated with the diseases to better understand the pathobiological mechanisms of these diseases [13, 14]. However, there is still a lack of the studies for multiple associated diseases prediction.

In recent years, neural network models have extensively been used for various NLP tasks, achieving competitive results [27–29]. The representative neural models include Convolutional Neural Network (CNN), Recurrent Neural Network (RNN), Long Short-Term Memory (LSTM) and Autoencoder, etc. Neural models are gradually applied in the tasks of biomedical field [30–34]. For example, Zhao et al., (2016) trained a deep multi-layer neural network model to extract protein-protein interactions information from biomedical literature [31]. However, neural networks have not been used for the task of multiple associated diseases prediction. In this paper, we explore a hybrid neural model for predicting kidney disease in hypertension patients.

## Methods

### Task modeling

When a patient is suffering from hypertension, the task aims to predict the probability of this patient who also has kidney disease. We model the prediction task based on the following steps.

1. We construct a dataset *D*^∗^ from the ground-truth EHR which contain these two diseases or only hypertension. Note that hypertension is labeled as $\mathcal {H}$, and kidney disease is labeled as $\mathcal {K}$. Specifically, positive examples indicate that patients suffer from both disease $\mathcal {H}$ and $\mathcal {K}$, which is denoted as *D*_+_∈*D*^∗^. Negative examples indicate that patients suffer from disease $\mathcal {H}$ but not $\mathcal {K}$, which is denoted as *D*_−_∈*D*^∗^.
2. At the training phase, we use the dataset *D*^∗^ that contains both *D*_+_ and *D*_−_ to train our model $\mathcal {M}$.
3. At the test phase, we apply the well-trained model $\mathcal {M}$ to predict a new EHR *d* of one patient, in which the diagnosis of $\mathcal {H}$ is confirmed, and the prevalence rate of $\mathcal {K}$ is to be inferred by $\mathcal {M}$.

### Neural network model

**Fig. 2:**
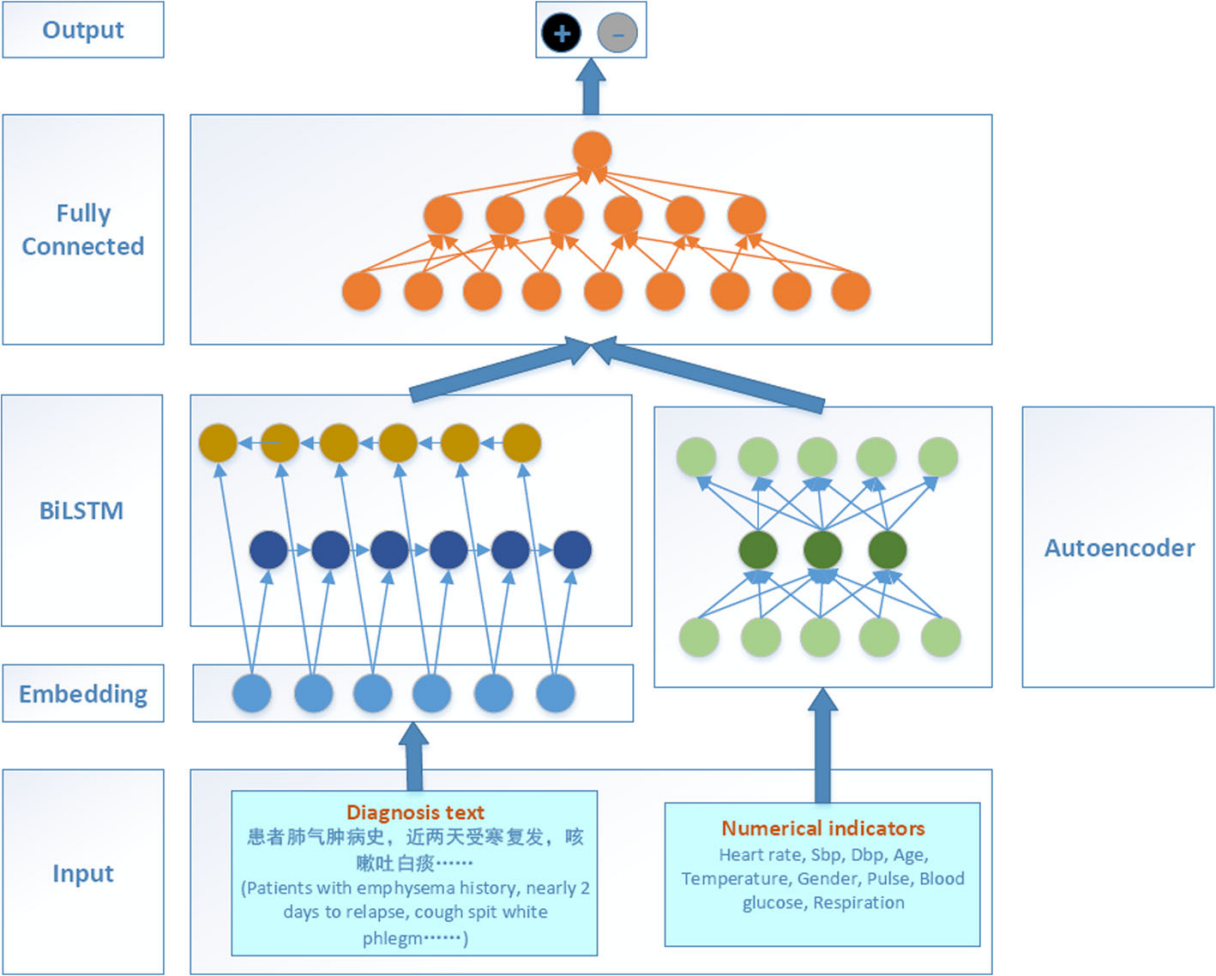
The proposed neural network framework

Figure 2 illustrates the proposed neural network, which includes two main parts: BiLSTM and Autoencoder. Here, BiLSTM is used for learning the continuous representation from the textual description information in EHR. Autoencoder is used for learning the continuous representation from the physical indicators in EHR.

#### Textual representation

The input from textual sentence describes the basic disease symptoms which may imply useful information behind the texts. We use an embedding layer to take the textual data as input. For each word or phrase *w*_*i*_, we use a look-up table *E* to obtain its embedding *e*(*w*_*i*_)∈*R*^*L*^, where *E*∈*R*^*L*×*V*^ is a parameter, *L* represents the dimension of embedding vector and *V* is the vocabulary size.

Then, a BiLSTM network is used to obtain the representation of each sentence. BiLSTM models a recurrent state transform sequence from an input sequence to a hidden state sequence. Basically, a LSTM represents each time step with an input, a memory and an output gate, denoted as *i*_*t*_,*f*_*t*_ and *o*_*t*_, respectively.

1 $$ \begin{aligned} i_{t}&= \sigma \left(W^{(i)}\right) x_{t} + U^{(i)} h_{i-1} + b^{i}\\ f_{t}&= \sigma \left(W^{(f)}\right) x_{t} + U^{(f)} h_{i-1} + b^{f} \\ o_{t}&= \sigma \left(W^{(o)}\right) x_{t} + U^{(o)} h_{i-1} + b^{o} \\ u_{t}&= tanh \left(W^{(u)}\right) + U^{(u)} h_{i-1} + b^{u}\\ c_{t}&= i_{t} \odot u_{t} + f_{t} \odot c_{t-1}\\ h_{t}&= o_{t} \odot tanh(c_{t})\\ \end{aligned}  $$

Where *σ* denotes the *sigmoid* function. Similar to the LSTM network, the architecture of BiLSTM network is designed to model the context dependency from the past and future. BiLSTM network has two parallel layers in both forward and backward directions, whose outputs is formulated as:

2 $$ \begin{aligned} h_{f_{t}}& = \sigma \left(W_{xh_{f}} x_{t} + W_{h_{f} h_{f}} h_{f_{t-1}} + b_{h_{f}}\right)\\ h_{b_{t}} &= \sigma \left(W_{xh_{b}} x_{t} + W_{h_{b} h_{b}} h_{f_{t-1}} + b_{h_{b}}\right) \\ \end{aligned}  $$

Here, the $h_{f_{t}}$ and $h_{b_{t}}$ denote the output of LSTM unit in forward layer and backward layer, respectively. We then concatenate these two hidden outputs as one total output:

3 $$ h^{(T)} = \left[h_{f_{t}};h_{b_{t}}\right]   $$

Based on the BiLSTM modeling, we obtain textual representation *h*^(*T*)^.

#### Numerical representation

For numerical features in our clinical data, since we replace the null values with the overall mean value, some of the values are correlated. Using these values directly may affect the performance of the task. Previous work shows that the denoising Autoencoder network can be utilized to reduce the high dimensionality and eliminate correlation [35]. Therefore, we employ this model to handle the numerical features.

Autoencoder is a network with multiple encoding layers, followed by one affine linear decoding layer. It maps the numerical values vector *v* into a hidden representation using an encoder function as follows:

4 $$ h^{(e)} = h\left(W^{(e) \cdot v + 1}\right)   $$

Then, a linear decoder reconstructs the hidden representation as follows:

5 $$ h^{(d)} = A \cdot h\left(W^{(d) \cdot h^{(e)} + 1}\right)   $$

where *A*=(*W*^2^)^*T*^ is a parameter, and *h* is ReLU function. Finally, we obtain a refined representation *h*^(*d*)^ of discrete physical values.

#### Outpur layer

A fully connected layer is used to combine two types of vectors from textual representation and numerical representation. This layer can be computed as:

6 $$ h^{(A)} = h\left(W^{(A)}\right) \cdot \left[ \begin{array}{c} h^{(T)}\\ h^{(d)} \end{array} \right]  $$

where *W*^(*A*)^ is a parameter, and *h* is ReLU function. Here, the dropout technique is utilized to avoid the overfitting. Finally, we employ the softmax activation function as the classifier in the bottom of the fully connected layer to obtain the output.

## Datasets

To construct the dataset of this task, we gather a large amount of EHR data, which is from the hospitals of 12 cities in China, with a span of 5 years ranging from 2012 to 2017. First, raw EHR data contains some personal privacy, e.g., patients’ name, resident ID number and institute number etc., so we remove these contents by pre-processing. Then, we merge records belonging to same patient into just one record. Specifically, a patient who suffers from different diseases receives more than one EHR with different diagnosis, but the physical indicators still keep same. Finally, we select a set of records from the merged EHR based on two criteria:

- A record where the patient suffers from both hypertension and kidney disease is selected as positive example.
- A record where the patient suffers from only hypertension is selected as negative example.

Based on the above steps, we get totally 35,332 records, in which 34,232 records are negative examples and 1100 records are positive examples. This is an extremely imbalanced dataset, and is problematic for directly conducting the experiments. To solve this problem, we employ undersampling method to balance the classes. Specifically, we decrease the size of majority class by randomly sampling a number of 1100 records in 34,232 records, so there are total 2200 examples in the dataset after undersampling, which is marked as *D*^∗^. In order to make full use of the dataset and make the result more credible, undersampling is repeated ten times. The final accuracy is the average result of the algorithms in all ten experiments.

## Experimental settings

We perform ten-fold cross-validation experiments and report the overall performances. The whole dataset is split into ten equal sections, each decoded by the model trained from the remaining nine sections. We randomly choose one section from the nine training sections as the development dataset in order to tune hyper-parameters. The classification result is measured by accuracy.

### Model parameters

There are two types of parameters in our experiments, including hyper-parameters and other settings. Specifically, *L* denotes the dimension of the word vectors, *L*_*BiLSTM*_ is the maximum length of the input textual sequences, *N*_*AE*_ is the number of Autoencoder layer, *N*_*MLP*_ is the number of fully connected layers. The dropout rate in fully connected layer is denoted as *R*_*dropout*_. *λ* is the initial learning rate for AdamGrad. In our model, the word embedding, *E*, is randomly initialized with uniform samples from $\left [-\sqrt {\frac {6}{r+c}}, +\sqrt {\frac {6}{r+c}}\right ]$, where *r* and *c* are the number of of rows and columns in the structure. Parameters are shown in Table 1.

**Table 1:** Parameter values of the model in the experiments

Parameter	*L*	*L*_*BiLSTM*_	*N*_*AE*_	*N*_*MLP*_
Value	100	128	6	2
Parameter	*λ*	*R*_*dropout*_	*batchsize*	*epochs*
Value	0.001	0.5	16	15

### Baselines

To demonstrate the effectiveness of the proposed algorithm, we re-implement the baseline systems which include discrete models and neural models. For each baseline model, we conduct the experiments by three types of inputs: 1) textual input only, note as Textual; 2) numerical input only, note as Numerical; 3) textual input and numerical input, note as Textual+Numerical.

**Discrete models**: Naive Bayes (NB), Support Vector Machine (SVM) and Gradient Boosting Decision Tree (GBDT) are used. These discrete models have extensively been used for text classification tasks, giving competitive results [36, 37]. Besides, Chen et al. (2017) explored the prediction problem of hypertension to coronary heart disease using Logistic Regression (LR) model combined with numerical physical indicators [15]. So we also use LR as a baseline.

**Neural models**: We use two neural models as neural baselines including Convolutional Neural Network (CNN) and Bi-directional Long Short Term Memory (BiLSTM). Besides, we integrate the Autoencoder (AE) with the neural model CNN as a hybrid model of CNN+AE, to make use of two types of features.

## Results

Based on the constructed dataset, Table 2 show experimental results of different discrete models. We can know that the LR model proposed by Chen et al. (2017) only gives 64.9% accuracy. The main reason is that this model only takes numerical physical indicators as input, ignoring the textual description information in EHR. This limits the performance of the task. By integrating the textual description information, the performance can be improved to 72.2% in accuracy. The NB model gives 76.6% accuracy based on Textual+Numerical features, outperforming the discrete LR model. This shows the effectiveness of the NB model in this task. Among all discrete models, SVM gives the relatively lowest results, giving 71.3% accuracy based on mixed features. The GBDT gives the highest accuracy (81.2%) based on Textual+Numberical features among all discrete models. The main reason is that GBDT is a boosting model which contains multiple meta classifiers and uses the assembling mechanism, and this makes GBDT model more powerful.

**Table 2:** Experimental results of the discrete baseline models

Models	Features	Accuracy(%)
LR	Numerical	64.9
LR	Textual	71.5
LR	Textual+Numerical	72.2
NB	Numerical	67.8
NB	Textual	71.1
NB	Textual+Numerical	76.6
SVM	Numerical	42.6
SVM	Textual	66.1
SVM	Textual+Numerical	71.3
GBDT	Numerical	71.1
GBDT	Textual	77.8
GBDT	Textual+Numerical	81.2

Table 3 shows the experimental results of different neural models. Among the neural baseline models, CNN achieves 86.2% accuracy on Textual+Numberical features. By integrating AE, CNN+AE achieves 88.3% accuracy on Textual+Numberical features, which is significantly higher than discrete models. This demonstrates that the neural network has powerful ability to fully learn the intrinsic features from the clinical data. Remarkably, the proposed BiLSTM+AE model gives the highest accuracy (89.7%) on Textual+Numberical features. Note that the Textual feature and Numerical feature achieve a slight lower score than the Textual+Numberical features. This indicates that two attention modules exert the role in improving the performance. The above analysis shows the effectiveness of the proposed neural model.

**Table 3:** Experimental results of the neural models

Models	Features	Accuracy(%)
CNN	Numerical	75.9
CNN	Textual	83.8
CNN	Textual+Numerical	86.2
BiLSTM	Numerical	74.8
BiLSTM	Textual	84.2
BiLSTM	Textual+Numerical	87.6
CNN+AE	Textual+Numerical	88.3
BiLSTM+AE	Textual+Numerical	89.7

Based on the above analysis, we can know that all model can give better performance based on the combination of textual and numerical features compared to the only textual features or numerical features. This is because different types of features in EHR data can both give their own contributions. Meanwhile, the results from only textual features are better than that from numerical features. The main reason is the textual description information intuitively carry strong cues for indicating a disease.

## Discussions

**Fig. 3:**
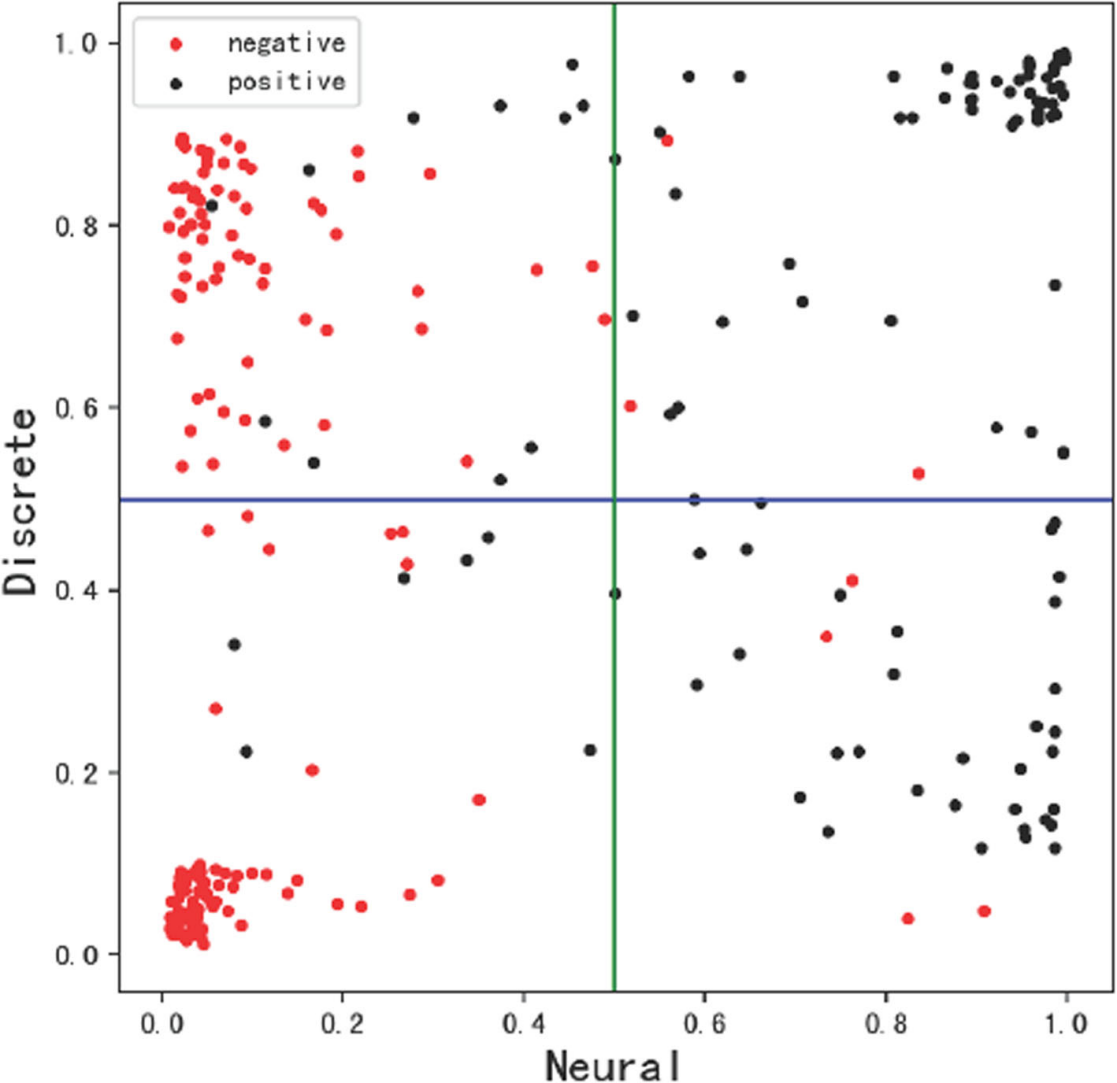
Comparison of output probability

We compare the output probability of the proposed neural model and the discrete model (GBDT) based on a test set to contrast the effect on discrete and neural features. Figure 3 shows the output probabilities of positive and negative classes. The x-axis shows the probability by the neural model and the y-axis shows the probability by the discrete model. The negative examples in the test set are shown in red, where positive examples are shown in black. As shown in the figure, most black dots are on the right of the figure and most red dots are on the left, showing that the results of neural model are correct in most cases. However, the dots are extremely more disperse in the y-axis and even many examples are wrongly scattered, which means that the discrete model is not very effective. This comparison demonstrates the effectiveness of our proposed neural model.

## Conclusions

We proposed a hybrid neural network model by integrating BiLSTM and Autoencoder networks for the prediction task of kidney disease in hypertension patients. Based on the constructed dataset from raw EHR data, the proposed model significantly outperform the current discrete model and the strong neural baseline systems.

In future, we will explore two directions. First, we will explore an attention-based neural network for the task. The attention mechanism can give different weights for different factors. We can visualize the risk factors for leading kidney disease in hypertension patients.. Second, we will study the problem of coronary heart disease prediction in hypertension patients, and shows the risk factors that cause hypertension patients develop into coronary heart disease.

## Abbreviations

AE: Autoencoder
BiLSTM: Bidirectional long short-term memory
CNN: Convolutional neural network
EHR: Electronic health records
GBDT: Gradient boosting decision tree
ICD-10: The 10th international classification of diseases
LSTM: Long short-term memory
NB: Naive Bayes
NLP: Natural language processing
RNN: Recurrent neural network
SVM: Support vector machine
